# ERBB2-modulated ATG4B and autophagic cell death in human ARPE19 during oxidative stress

**DOI:** 10.1371/journal.pone.0213932

**Published:** 2019-03-14

**Authors:** Shwu-Jiuan Sheu, Jiunn-Liang Chen, Youn-Shen Bee, Shi-Han Lin, Chih-Wen Shu

**Affiliations:** 1 Department of Ophthalmology, Kaohsiung Veterans General Hospital, Kaohsiung, Taiwan; 2 School of Medicine, National Yang-Ming University, Taipei, Taiwan; 3 Department of Optometry, Shu-Zen Junior College of Medicine and Management, Kaohsiung, Taiwan; 4 Yuh-Ing Junior College of Health Care and Management, Kaohsiung, Taiwan; 5 National Defense Medical Center, Taipei, Taiwan; 6 School of Medicine for International Students, I-Shou University, Kaohsiung, Taiwan; 7 Institute of Biomedical Sciences, National Sun Yat-Sen University, Kaohsiung, Taiwan; Univerzitet u Beogradu, SERBIA

## Abstract

Age-related macular degeneration (AMD) is an ocular disease with retinal degeneration. Retinal pigment epithelium (RPE) degeneration is mainly caused by long-term oxidative stress. Kinase activity could be either protective or detrimental to cells during oxidative stress; however, few reports have described the role of kinases in oxidative stress. In this study, high-throughput screening of kinome siRNA library revealed that erb-b2 receptor tyrosine-protein kinase 2 (ERBB2) knockdown reduced reactive oxygen species (ROS) production in ARPE-19 cells during oxidative stress. Silencing ERBB2 caused an elevation in microtubule associated protein light chain C3-II (MAP1LC3B-II/I) conversion and sequesterone (SQSTM)1 protein level. ERBB2 deprivation largely caused an increase in autophagy-regulating protease (ATG4B) expression, a protease that negatively recycles MAP1LC3-II at the fusion step between the autophagosome and lysosome, suggesting ERBB2 might modulate ATG4B for autophagy induction in oxidative stress-stimulated ARPE-19 cells. ERBB2 knockdown also caused an accumulation of nuclear factor erythroid 2-related factor 2 (NRF2) and enhanced its transcriptional activity. In addition, ERBB2 ablation or treatment with autophagy inhibitors reduced oxidative-induced cytotoxic effects in ARPE-19 cells. Furthermore, ERBB2 silencing had little or no additive effects in ATG5/7-deficient cells. Taken together, our results suggest that ERBB2 may play an important role in modulating autophagic RPE cell death during oxidative stress, and ERBB2 may be a potential target in AMD prevention.

## Introduction

Age-related macular degeneration (AMD) is one of the most common diseases that cause uncorrectable severe vision loss in elder people worldwide [1]. AMD is also a retinal degenerative disease and the main cause of visual acuity and color vision. AMD can be categorized into several groups, depending on histopathological features. Drusen is caused by protein and lipid accumulation in retinal pigment epithelium (RPE) and Bruch’s membrane of patients with early and intermediate AMD then become advanced AMD. Advanced AMD is further categorized as geographic atrophy (GA) or neovascular AMD (NVAMD or wet/exudative AMD). GA and early and intermediate AMD are normally considered as dry AMD [2], whereas AMD with choroidal neovascularization is referred to as wet/exudative AMD. Patients with early and intermediate AMD present few effects with respect to visual acuity impairment, and advanced AMD may cause blindness [3, 4].

While photoreceptor death in the central retina is involved in vision loss in AMD patients, early pathogenesis may result from degeneration of the RPE, a pigmented ciliated epithelial cell. RPE cells reportedly undergo apoptosis, a type I programed cell death, in AMD eyes [5, 6]. Due to its juxtaposition to the choriocapillaris, which is in a high blood stream with high oxygen, RPE cells are exposed to high oxygen microenvironment [6]. While AMD pathophysiology is not fully understood, these studies have implicated oxidative damage in AMD pathogenesis [7]. Epidemiological studies also show that smoking is positively associated with AMD, whereas an antioxidant diet was reported to reduce risk of progression to advanced AMD [8].

Kinases act as upstream regulators in signaling pathways in order to maintain cellular homeostasis in normal conditions and lead to cell death in response to various stresses, including oxidative stress. The vascular endothelial growth factor (VEGF) gene locus is highly associated with both wet and dry AMD [9]. Elevated VEGF levels trigger IL-1β activation of inflammation via cryopyrin (NRLP3)-mediated inflammasome formation [10]. Oxidative stress induces the mammalian target of rapamycin (mTOR) activation involved in RPE cell differentiation and hypertrophy, which in turn initiates photoreceptor degeneration [11]. Several kinase inhibitors against VEGF and mTOR have been proposed as therapeutic treatment for AMD (ClinicalTrials.gov identifier: NCT00304954). However, the effects of other kinases on the response of RPE cells to oxidative damage remain unknown. In this study, we conducted kinome-wide siRNA screening for potential kinase targets that may be required for oxidative stress-induced cytotoxicity of RPE cells. The results show that silencing the erb-b2 receptor tyrosine-protein kinase 2 (ERBB2) offered protection from oxidative damage-associated oxidative stress, which might involve activation of autophagy-regulating protease (ATG4B) and nuclear factor erythroid 2-related factor 2 (NRF2) and a diminution in autophagy. Our findings suggest that ERBB2 might be a potential marker or therapeutic target for AMD patients.

## Material and methods

### Reagents and cell culture

Hydrogen peroxide (H_2_O_2_) 35% was purchased from Sigma-Aldrich (349887, Merck KGaA, USA). Dulbecco’s modified Eagle’s medium (DMEM) and Ham’s F12 medium were obtained from GIBCO (Life Technologies; Carlsbad, USA). CellTiter-Glo assay (G7572), Nano-Glo luciferase and ROS-Glo Hydrogen Peroxide assay kits were purchase from Promega Corporation (Madison, WI, USA). Chloroquine (CQ; Sigma-Aldrich, C6628) and Concanavalin A (ConA, MERCK, MO, 344085) were dissolved in dimethyl sulfoxide (DMSO) to prepare stock solutions. Human RPE cell cultures (ARPE-19) were purchased from the American Type Culture Collection (CRL-2302; ATCC) and cultured as previously described [12].

### Cell viability assay

ARPE19 cells were seeded at 5000 cells/well in 96-well plates and either silenced with siRNA against ERBB2 (Ambion, s611 or Dharmacon, 2064), ATG5 (Dharmacon, 9474), ATG7 (Ambion, s20652), unc 51 like autophagy kinase 1(ULK1) (Dharmacon, 8408), or beclin 1 (BECN1) (Ambion, s16538) or treated with 20 μM CQ and ConA. Cell viability was measured by CellTiter-Glo Luminescent Assay kit (G7572) according to the manufacturer’s instructions. The method allows detection of cellular ATP level via generation of a luminescent signal.

### Luminescent ROS and NRF2 reporter assay

ARPE19 cells were seeded in 384-well plates containing RNAimax (Invitrogen, 13778–150) and pooled siRNA library against human kinase or kinase related genes (709 genes, A30079, Thermo Fisher Scientific) for 48 h. The transfected cells were exposed to hydrogen peroxide for 24 h. The medium was replaced with fresh culture medium, and intracellular ROS levels were assessed using the ROS-Glo Hydrogen Peroxide Assay kit (G8820) according to the manufacturer’s instructions. The assay utilizes a derivatized luciferin substrate that reacts with hydrogen peroxide to produce a luciferin product, generating a luminescent signal that allows quantification of hydrogen peroxide in the cell. All experiments were repeated at least three times. For the NRF2 reporter assay, cells were transfected with vector (PGL 4.37) harboring the NRF2-binding promoter (AGCTTGGAAATGACATTGCTAATGGTGACAAAGCAACTTTTAGCTTGGAAATGACATT GCTAATGGTGACAAAGCAACTTT) for luciferase expression. One-Glo was added to the treated cells, and NRF2 activity was measured with a luminescent reader.

### Real-time PCR

ARPE-19 cells transfected with siRNA were used for the extraction of total RNA with TRIzol Reagent (Invitrogen, 15596–018). 1 μg RNA was converted to cDNA with Reverse Transcriptase (Invitrogen, 18064–014), and ToolsQuant II Fast RT kit (Tools, Taiwan, KRT-BA06) was used for cDNA synthesis. The amount of target gene mRNA, hemoxygenase (HO)-1 relative to glyceraldehyde 3-phosphate dehydrogenase (GAPDH) was analyzed using a real-time polymerase chain reaction (qPCR) performed in the StepOnePlus system (Applied Biosystems) using SYBR Green Master Mix (Applied Biosystems, 4385612). The primers for the qPCR will be provided upon request.

### Immunoblotting

Cells were harvested and lysed followed by immunoblotting for detection of protein levels as previously described [13]. Briefly, the cells were lysed with radioimmunoprecipitation assay (RIPA) buffer containing protease inhibitor cocktail (Roche, 11873580001). Cellular proteins were separated with sodium dodecyl sulfate-polyacrylamide gel electrophoresis (SDS-PAGE) and transferred onto nitrocellulose membranes. The proteins on the membrane were probed with primary antibodies against MAP1LC3B (Sigma-Aldrich, L7543) and ACTB (β-actin, Sigma-Aldrich, A5441); sequesterone (SQSTM1) (BD Pharmingen, 610832), ERBB2 (Cell Signaling Technology, 29D8), ULK1 (Cell Signaling Technology, D8H5), ATG5 (Cell Signaling Technology, 8540), and ATG7 (Cell Signaling Technology, 8558), NRF2 (Santa Cruz, sc-13032 or Abcam, ab137550) and BECN1 (SC-11427) (Santa Cruz), and then detected with an HRP-labeled secondary antibody (Santa Cruz, sc-2005). For cell fractionation, collected cells were incubated with 200 μl lysis buffer containing 0.5% NP40 and protease cocktail (Sigma, Cat. no. P-2714) on ice for 15 min in order to break cell membranes followed by centrifugation at 1300 xg for 10 min to collect nuclear fractions. The supernatant was further centrifuged at 14,000 xg for 30 min in order to harvest cytoplasmic fractions. Equal amounts of 2X Laemmli SDS-sample buffer was used to lyse both the nuclear pellet and cytoplasm for further immunoblotting. For the *in vitro* cleavage assay as reported formerly [14], proteins were incubated with 250 nM protein substrates (LC3B-Stag, GATE-Stag) in 50 μl reaction buffer containing 50 mM Tris (pH 8.0), 1 mM dithiothreitol, and 150 mM NaCl at 37°C for 2 h. Fifty microliters of 2X Laemmli SDS-sample buffer was used to stop the reaction for immunoblot analysis using anti-S-tag antibody (Novogen, 71549) or anti-myc antibody (Roche, 11667203001).

### Transfection and infection

For siRNA transfection as described previously [15], cells were reversely transfected with RNA iMAX (Life Technologies; 13778–150) and 5 nM scramble siRNA (Life Technologies, 12935–112), kinome siRNA library (2127 siRNA for 709 gene, A30079, Thermo Fisher Scientific), ATG5 (GE Healthcare Dharmacon, 9474), ATG7 (Life Technologies, s20652), BECN1 (Life Technologies, 4392420), ULK1 (Dharmacon, 8408), or ERBB2 (Life Technologies, 4390824;) for 72 h. For shRNA infection, 8XARE reporter plasmid, a packaging and VSV-G expressing envelope plasmid, were transfected into HEK293T cells using the transfection reagent, Lipofectamine 2000 (Life Technologies, 11668–027) for two days to achieve an infection. Cells were then harvested for further experiments or to confirm knockdown efficiency via immunoblotting.

### Statistical analysis

All data were expressed as the mean ± standard error of the mean (SEM) of at least three independent experiments within one month to avoid the huge difference caused by different cell passages. The Mann-Whitney U-test was used to perform statistical analysis with Prism 5.0 (Graph-Pad) to compare the effects between each group. Significant results were marked as *P <0.05, **P <0.01 or *** *P* <0.001.

## Results

**Fig 1:**
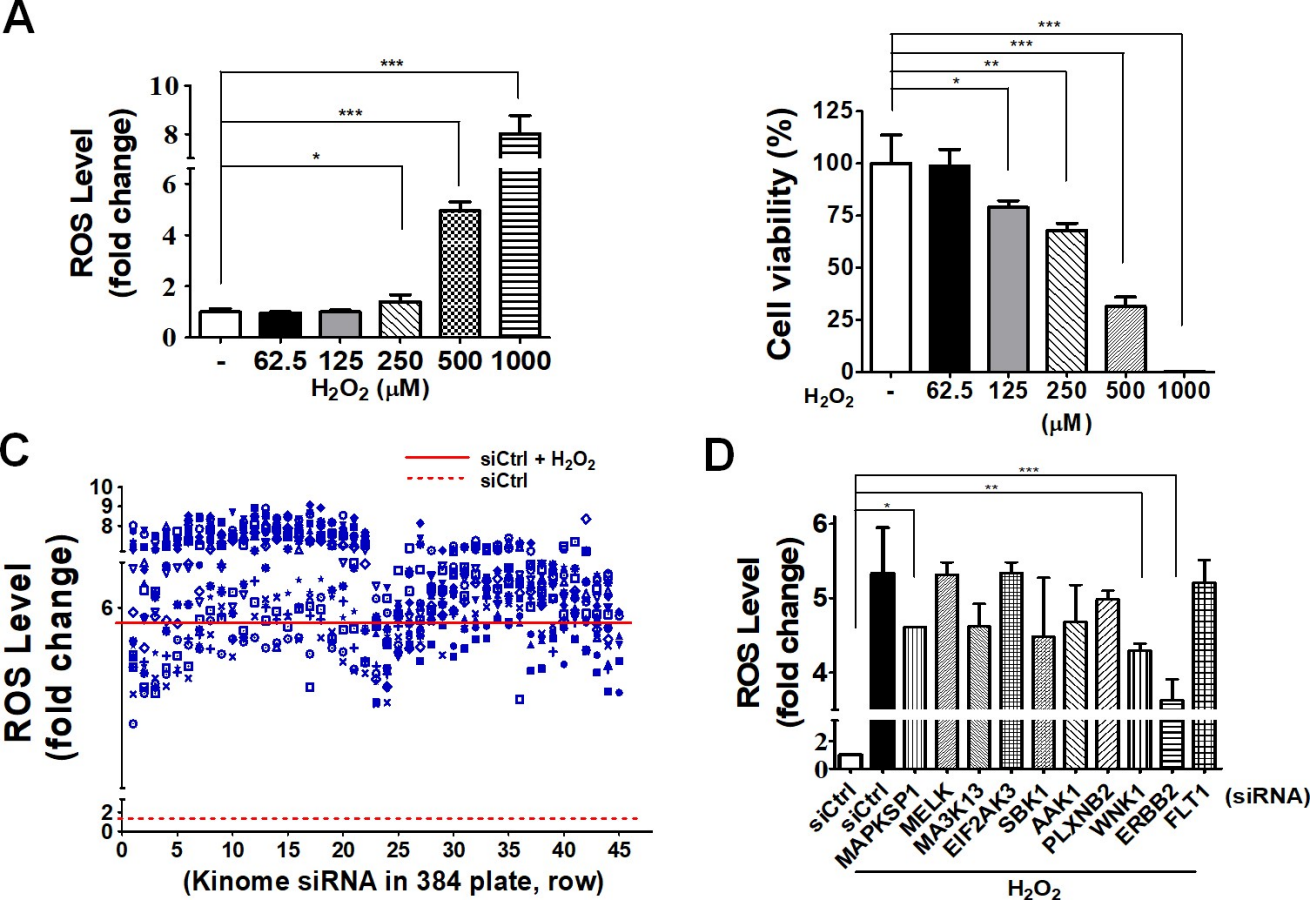
Kinome siRNA screening for cytotoxic effects of ARPE-19 cells during oxidative stress. (A) Human RPE ARPE-19 cells were treated with non-targeting siRNA for 48 h, followed by treatment with hydrogen peroxide at 62.5, 125, 250, 500, and 1000 μM for 24 h in order to determine cell viability. Cellular ROS production and (B) cell viability were measured with ROS-Glo and Cell-titer Glo, respectively. (C) Cells were treated with kinome siRNA (710 gene) for 48 h followed by treatment with hydrogen peroxide (500 μM) for 24 h in order to measure ROS production in cells. (D) The top 10-ranked hits from kinome siRNA screening were further validated for cellular ROS production in three independent experiments (Three parallel samples were included in each experiment), and the results are shown as mean ± SEM.

In order to screen potential kinase involvement in oxidative stress-induced ROS production and cell death, we first optimized ROS production in ARPE-19 cells during oxidative stress. ARPE-19 cells were transfected with scramble siRNA and then exposed to various concentrations of hydrogen peroxide (62.5, 125, 250, 500, 1000 μM). The ROS level significantly increased in ARPE-19 cells in a dosage-dependent manner (Fig 1A). We also observed that at the highest concentration of hydrogen peroxide, ARPE-19 cell viability was markedly reduced (Fig 1B). The signal-to-noise ratio was >3 using hydrogen peroxide at 500 μM; therefore, this concentration was selected for conducting high throughput screening. Using a kinome siRNA library consisting of 710 kinase-related genes, kinome-wide siRNA screening was conducted to identify potential kinases that may promote intrinsic ROS production in cells under oxidative stress (Fig 1C), and the top 10 hits were selected for further validation (Fig 1D). Knockdown of three candidate genes, mitogen-activated protein kinase (MAPK)SP1, serine/threonine protein kinase (WNK1), and receptor protein tyrosine kinase (ERBB2) caused a reduction in ROS production in ARPE-19 during oxidative stress (Fig 1D).

**Fig 2:**
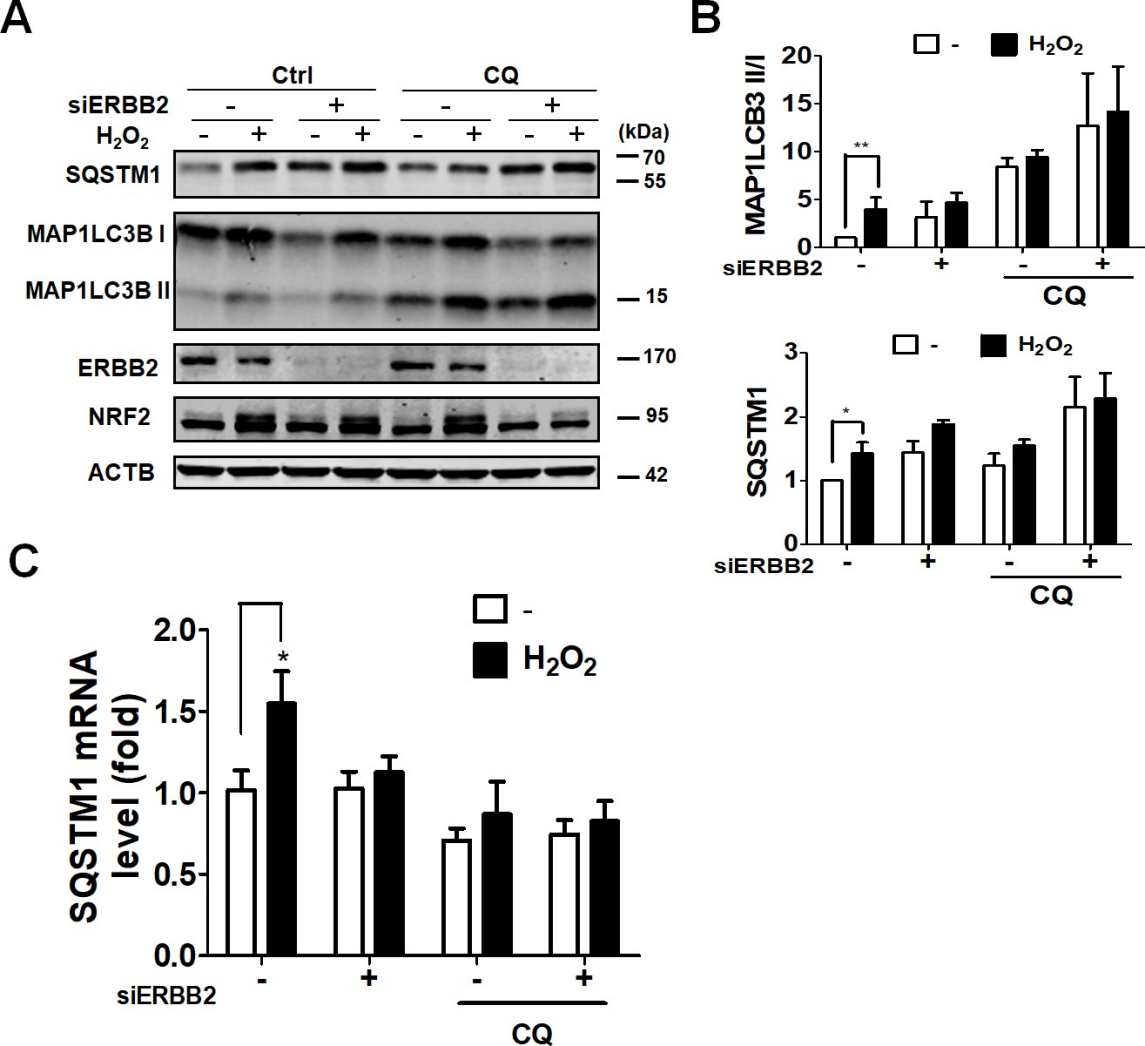
Effects of ERBB2 on autophagy in ARPE-19 cells during oxidative stress. (A) Human RPE ARPE-19 cells were transfected with 5 nM scramble siRNA or siRNA against ERBB2 for 64 h and then treated with hydrogen peroxide (500 μM) for 8 hr. The cells were lysed for western blotting using antibodies against NRF2, SQSTM1, MAP1LC3B, ATG4B, and ACTB. (B) The quantitative results for ratio of MAP1LC3B-II/I and SQSTM1 protein level are shown. (C) The mRNA levels of SQSTM1 in cells as mentioned above were determined by real-time polymerase chain reaction (PCR). The results were analyzed using Prism 5.0 and expressed as mean ± SEM from three independent experiments (Three parallel samples were included in each experiment).

NRF2 and autophagy are critical for inhibiting intracellular ROS production. Autophagy induction triggers degradation of kelch-like ECH-associated protein 1 (KEAP1), which is a substrate adaptor protein for the Cullin3 (Cul3)-containing E3-ligase complex. KEAP1 degradation further liberates NRF2 to activate SQSTM1 expression [16]. Accordingly, we evaluated NRF2 and autophagy involvement in ARPE-19 cells during oxidative stress. Hydrogen peroxide induced conversion of MAP1LC3B-I to MAP1LC3B-II and caused an increase in SQSTM1 protein level (Fig 2A and 2B). Silencing of ERBB2 or treatment with autophagy inhibitor CQ also caused an increase in SQSTM1 protein level, which was further augmented with hydrogen peroxide co-treatment (Fig 2A and 2B). Hydrogen peroxide also causes an elevation in SQSTM1 mRNA levels, whereas silencing ERBB2 and treatment with CQ diminished the hydrogen peroxide-induced elevation of SQSTM1 mRNA level (Fig 2C). These results suggest that hydrogen peroxide may transcriptionally activate SQSTM1 gene expression, whereas ERBB2 knockdown may inhibit autophagy and allow intracellular accumulation of SQSTM1 during oxidative stress.

**Fig 3:**
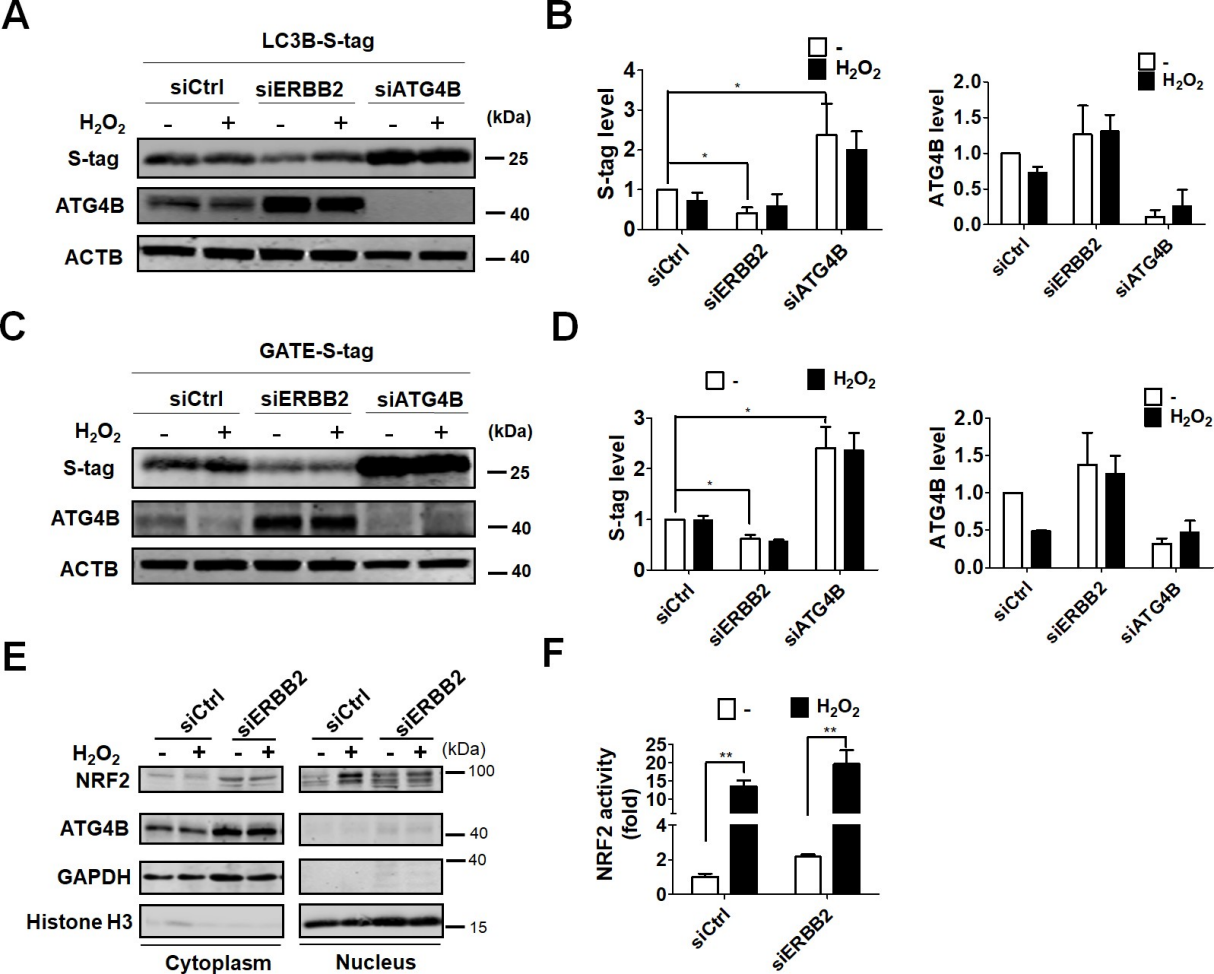
Effects of ERBB2 on ATG4B in ARPE-19 cell during oxidative stress. Cells were transfected with 5 nM scramble siRNA or siRNA against ERBB2 or ATG4B for 48 h, followed by treatment with hydrogen peroxide (500 μM) for 24 h. The cells were then lysed, and equal amount of proteins were incubated with S-tagged (A) MAP1LC3B and (C) GATE-16 for 2 h. S-tag removal and ATG4B expression were examined by immunoblotting (B and D). The S-tag and ATG4B protein levels were quantitated with image J and expressed as mean ± SEM. (E) The knock-downed cells in the absence or presence of hydrogen peroxide were harvested, and nuclear and cytoplasmic fractions were split. The fractionated proteins were determined by immunoblotting using antibodies against NRF2 and ATG4B. (F) NRF2 transcriptional activity was monitored in cells harboring vector containing NRF2 promoter and luciferase.

ATG4B is a key protease required for pro-MAP1LC3B activation and MAP1LC3B-II deconjugation. ROS has been shown to spatiotemporally inactivate ATG4 to promote autophagy in starved cells [17]. Indeed, silencing ATG4B elevates autophagic flux in colorectal cancer cells, which implies that excessive ATG4B has negative effects on autophagy [18]. In order to examine the involvement of ATG4B expression and activation in ERBB2-modulated autophagy in ARPE-19 cells, silenced cell lysates were incubated with S-tagged recombinant substrates, including MAP1LC3B and GATE16, and we found that knockdown of ERBB2 elevated ATG4B protein level. Removal of S-tag from the C-terminus of MAP1LC3B or GATE16 was also enhanced in ERBB2-silenced cells, whereas knockdown of ATG4B let of accumulation of S-tagged MAP3LC3B and Golgi-associated ATPase enhancer (GATE16) (Fig 3A–3D), suggesting that ERBB2 may inactivate ATG4B to facilitate autophagy in cells under oxidative stress. Similar to treatment with hydrogen peroxide, ERBB2 knockdown caused an elevation in nuclear localization of NRF2 (Fig 3E) and its transcriptional activity (Fig 3F), which provides clues to the way in which silencing ERBB2 reduces ROS levels in ARPE-19 cells during oxidative stress.

**Fig 4:**
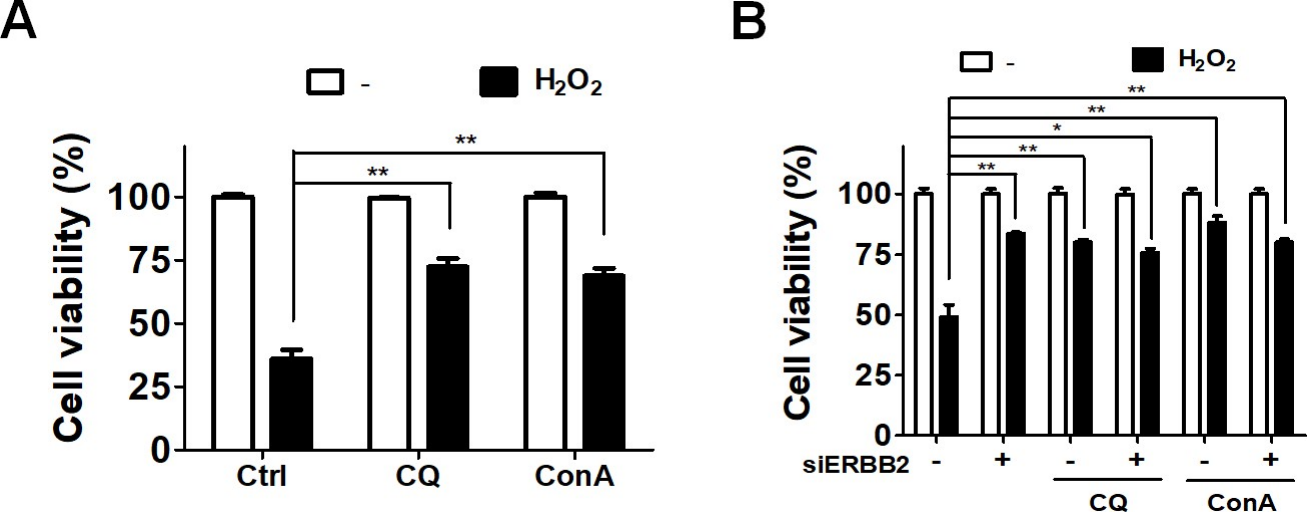
Effects of autophagy inhibitors in oxidative stress-induced cell death. (A) Human RPE ARPE-19 cells were treated with hydrogen peroxide (500 μM) in the absence or presence of autophagy inhibitor CQ (20 μM) or ConA (10 nM) for 24 h. Cell viability was quantified with Cell-titer Glo assay system. (B) The cells were transfected with 5 nM scramble siRNA or siRNA against ERBB2 for 48 h and treated with hydrogen peroxide (500 μM) in the absence or presence of autophagy inhibitors CQ (20 μM) or ConA (10 nM) for 24 h. The results were analyzed with Prism 5 and expressed as mean ± SEM from three independent experiments (Three parallel samples were included in each experiment).

**Fig 5:**
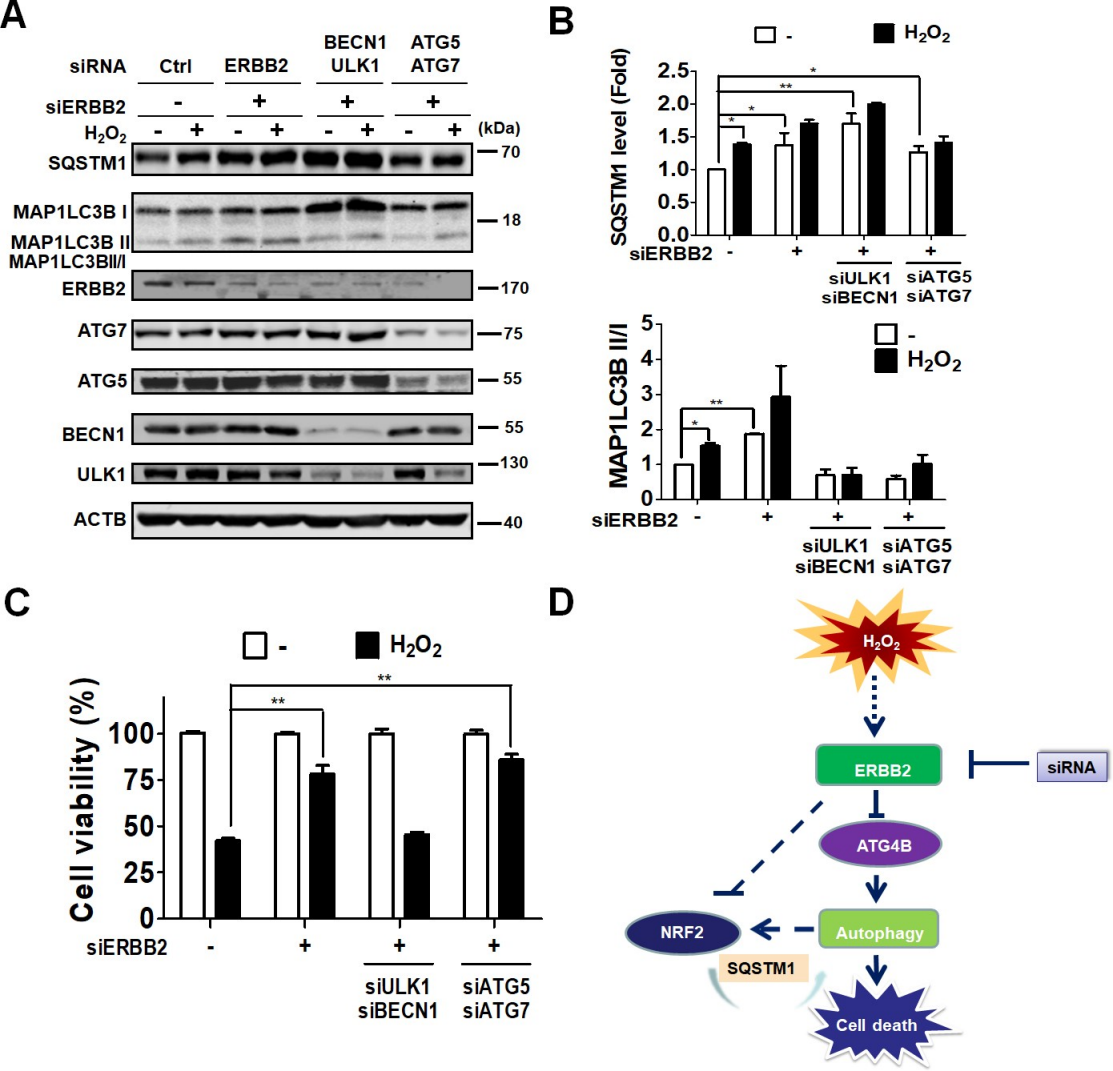
Effects of ERBB2 in autophagy deficient ARPE-19 cells during oxidative stress. (A) Human RPE ARPE-19 cells were transfected with 5 nM scramble siRNA or siRNA against ERBB2 without or with ULK1, BECN1, ATG5, and ATG7 for 48 h and treated with hydrogen peroxide (500 μM) for 24 h. The cells were lysed for immunoblotting to determine protein level of ERBB2, ATG5, ATG7, BECN1, ULK1, SQSTM1, and MAP1LC3B using ACTB as the internal control. (B) SQSTM1 protein levels and ratio of MAP1LC3B-II/I were quantitated with image J and expressed as mean ± SEM. (C) The knock-downed cells were treated with hydrogen peroxide (500 μM) for 8 h, and cell viability was quantified with Celltiter-Glo assay system. (D) Schematic diagram for the potential role of ERBB2 in autophagic cell death in ARPE-19 cells during oxidative stress.

Autophagy could play a protective or detrimental role in cells during periods of stress. In order to further inspect the role of autophagy and ERBB2 in oxidative stress-induced cell death, ARPE-19 cells were treated with hydrogen peroxide in the absence or presence of autophagy inhibitors, including CQ and ConA (Fig 4A), and we found that inhibition of autophagy significantly reduced oxidative stress-induced cell death. Likewise, knockdown of ERBB2 caused an increase in the number of viable cells during oxidative stress (Fig 4B). Furthermore, ATG5 and ATG7 are essential genes required for autophagy, while ULK1 and BECN1 are genes involved in ATG5/7-independent autophagy. Deprivation of ERBB2 or addition of siRNA against ATG5/7 or ULK1/BECN1 resulted in accumulation of SQSTM1 (Fig 5A and 5B). Interestingly, silencing AERBB2 in ATG5/7-deficient cells significantly recovered cell viability during oxidative stress (Fig 5C). However, ERBB2-silenced cells acquired no protective effects from ULK1/BECN1 knockdown compared to cells harboring siRNA against ERBB2 alone. These results suggesting knockdown of ULK1/BECN1 may have additional cytotoxic effects in ARPE19 cells. Taken together, hydrogen peroxide may induce autophagic cell death in ARPE19 cells via ERBB2 modulation. Ablation of ERBB2 or the autophagic process protects cells from oxidative stress as illustrated in the schematic diagram in Fig 5D.

## Discussion

Oxidative stress has been shown to be highly associated with AMD development of AMD. It has been suggested that antioxidants and kinases may aid in preventing AMD; however, the molecular mechanisms that confer protection against oxidative stress is not clear. Our study involving kinome siRNA library screening revealed a number of interesting findings. First, silencing of ERBB2 inhibited ROS production in ARPE-19 cells during oxidative stress. Second, deprivation of ERBB2 resulted in induction of autophagy and NRF2 activation, most likely due to elevated ATG4B expression. Third, knockdown of ERBB2 might reduce autophagic cells death in ARPE-19 cells during oxidative stress. Overall, our results suggest that in ARPE-19 cells, ERBB2 may cause a ATG4B diminution that promotes autophagic cell death with NRF2 inactivation in response to oxidative stress.

ERBB2 is a tyrosine kinase that belongs to the epidermal growth factor receptor (EGFR) family, which include ERBB1 (EGFR/HER1), ERBB2 (HER2), ERBB3 (HER3), and ERBB4 (HER4) [19–21]. Among the four members, ERBB2 has the highest kinase activity, and upon dimerization with ERBB3, forms a heterodimer with the strongest signaling function [22]. In breast cancer, ERBB2 over-expression is highly associated with tumorigenesis. ERBB2 expression levels serve as a biomarker for targeted therapy. In regard to autophagy, ERBB members could induce or inhibit autophagy in a context-dependent manner [23]. ERBB1 phosphorylates and activates AKT, which in turn activates mTOR, an autophagy inhibitor that also inhibits autophagy modulation via ULK1 activation [22, 24]. Moreover, ERBB1 attenuates autophagy by both reducing BECN1 and vps34 association in to enhancing BCL-2 binding to BECN1 [25]. On the other hand, recruitment of lysosomal-associated transmembrane protein 4B (LAPTM4B), Sec5, and RUN domain protein as Beclin 1-interacting and cysteine-rich containing (Rubicon) by ERBB1 liberates BECN1 for autophagy induction [26], a process that is independent of kinase activity. ERBB2 over-expression was shown to suppress stress-induced autophagy [27]. Our present study showed that ERBB2 may cause a reduction in ATG4B expression and MAP1LC3-II deconjugation to promote autophagy in ARPE-19 cells during oxidative stress, suggesting varied involvement of different ERBB isoforms in autophagy modulation. Several kinase inhibitors that negatively regulate autophagy have been proposed for use as therapeutic treatment for AMD, including anti-VEGF agents and mTOR inhibitors. Nevertheless, ERBB2 may be the link between kinases and autophagy in RPE cells, at least in the context of oxidative damage, and further studies are warranted in order to elucidate the relationship.

Hydrogen peroxide oxidizes the cysteine residue of AMPK and ATG4, leading to autophagy induction. Post-translationally, hydrogen peroxide modifies α and β subunits of adenosine monophosphate-activate protein kinase (AMPK) to increase its kinase activity in human embryonic kidney (HEK)293T cells [28], which facilitates autophagy via ULK1 activation and mTOR inactivation [29, 30]. Furthermore, ATG4 modulates autophagy through involvement in the conjugation and deconjugation of ATG8 homologues, including MAP1LC3 and gamma-aminobutyric receptor-associated protein (GABARAP) family members [31]. ATG4 initially cleaves the C-terminus of MAP1LC3 to expose the C-terminal glycine residue for conjugation with phosphatidylethanolamine (PE) and thus leads to autophagosome formation [17]. The PE-conjugated MAP1LC3 (MAP1LC3-II) can be cleaved by ATG4 and released from the autophagosome [28], which is likely a critical step for efficient autophagy. Hydrogen peroxide spatiotemporally inactivates ATG4 to ensure autophagosome formation and lysosome fusion in cells during starvation [17]. In addition, phosphorylation of ATG4B is elevated to enhance ATG4B activity in cells during autophagy-inducing conditions [32]. We found that ERBB2 modulated ATG4B levels and activity in ARPE-19 cells during oxidative stress. Nevertheless, the detailed mechanisms of ERBB2 involvement in ATGB4 phosphorylation and oxidization require further studies.

Autophagy is induced in cells in response to oxidative stress via several factors such as protein kinase R-like endoplasmic reticulum kinase (PERK), hypoxia-induced factor 1(HIF1), p53, NRF2, and forkhead box (FOX)O3 [33]. Moreover, autophagy can reduce oxidative stress through the pathway involving NRF2/KEAP1 and SQSTM1/p62 [34, 35]. SQSTM1/p62, an autophagy substrate and cargo adapter, can interact with and target KEAP1 for selective degradation by autophagosomes [36], thus releasing NRF2. NRF2 is a transcription factor of the leucine zipper family and can activate genes for antioxidant-defense proteins such as glutathione peroxidase, superoxide dismutase, and thioredoxin [37]. Under normal conditions, NRF2 is sequestered by KEAP1 and inactivated by proteasomal degradation. Following oxidative stress, either KEAP1’s ubiquitin activity is inhibited or SQSTM1/p62 interacts with KEAP1 leading to NRF2 liberation and activation, which in turn limits ROS production and cell damage. However, ROS also down-regulates ULK1 to inhibit autophagy via phosphorylation of p53 in NB4 cells [38]. These findings suggest that the effects of ROS on autophagy may depend on dosage and time of ROS exposure or on cell type. In this study, although NRF2 might be involved in SQSTM1 expression for autophagy induction, the effect was relatively weak (Fig 2, ~1.6-fold increase) compared NRF2’s transcriptional activity (Fig 3, ~20-fold increase). We think that ERBB2 may positively modulate autophagy, whereas it may have an autophagy-independent root for negatively modulating NRF2 in cells during oxidative stress. Silencing ERBB2 might trigger both NRF2 activation and autophagy induction. Moreover, NRF2 is a transcriptional factor for several antioxidant genes, which may reduce ROS production as shown in Fig 1. However, since the protective effects of knockdown of ERBB2 are similar to autophagy inhibitory cells during oxidative stress, ERBB2-modulated autophagy might be detrimental in cells during oxidative stress.

Autophagy has been implicated as a protective pathway for stress-induced injury prevention, mainly through damaged protein and organelle removal in cells. Autophagy is elevated and plays a cytoprotective role in several ocular diseases such as herpes simplex virus type 1 (HSV-1) infection, cataracts, glaucoma, diabetic retinopathy (DR) [39, 40]. However, increasing evidence implicates autophagy in stress-induced cell death in certain settings. For example, autophagy has been found to be elevated in ischemia-induced neuronal cell death in the hippocampus [41]. Deprivation of autophagy with either inhibitor 3-methyladenine or RNAi against BECN1 or ATG7 protects neurons from ischemia-induced death. Cardiac injury caused by ischemia reperfusion is also diminished in BECN1 heterozygous mice [42]. Moreover, hydrogen peroxide activates several autophagy-regulating proteins to promote autophagic cells death [43]. In line with our study, ERBB2 is involved in autophagic cell death resulting from oxidative stress, which is the main cause of AMD. These finding support ERBB2 as a potential target in the prevention and treatment of AMD.

## Abbreviations

MAP1LC3B: microtubule associated proteins 1A/1B light chain 3B
SQSTM1: sequestosome 1
ATG: autophagy-related
BECN1: beclin 1
CQ: chloroquine
RPE: retinal pigment epithelium
ROS: reactive oxygen species
